# Supplementary material for: Livestock grazing is associated with seasonal reduction in pollinator biodiversity and functional dispersion but cheatgrass invasion is not: Variation in bee assemblages in a multi-use shortgrass prairie
Source: PLoS One. 2020 Dec 17;15(12):e0237484. doi: 10.1371/journal.pone.0237484 (PMC7746148; doi:10.1371/journal.pone.0237484)
Supplement: S1 Table — Functional trait values were determined from data or information found in Michener [33, 35, 37–40]. (DOCX) [file pone.0237484.s001.docx]

| **Trait** | **Categories or values** | **Description and sources** |
| --- | --- | --- |
| Lecty (dietary specialization) | 1: Oligolectic  2: Polylectic  3: Kleptoparasite | Oligolectic are bee species forage on one to four forb genera. Polylectic are bees forage on five or more genera (Scott et al. 2011) (Murray et al. 2009). Species with no evident lecty status are kleptoparasites; these species may be oligolectic or polylectic depending on the diet breadth of species they parasitize. |
| Nesting construction | 1: Excavators  2: Rent  3: Kleptoparasite | For excavator species female bees excavate their own nests. Renters inhabit pre-existing cavities or nesting spaces (Potts et al. 2005). Kleptoparasites rob the nests of other bees and nest construction related to the nesting habits of species they parasitize. |
| Nesting location | 1: Above ground  2: Below ground  3: Kleptoparasite | Bees living above ground are above-ground nesters, while those bees which nest below ground are below-ground nesters. Kleptoparasites may inhabit either type of nest as they exploit nests already constructed by other bees. |
| Tibia hair density | 1: High  2: Medium  3: Low | High tibial hair density was defined by setae density that totally obscures viewing the tibia. Medium tibial hair density was defined as dense setae but able to view the tibia. Low tibia hair density was defined as sparse or intermittent setae with a corbicula. |
| Tibia pollen collecting structure | 1: Corbicula  2: Scopa  3: Abdomen scopa  4: Kleptoparasite | A corbicula is a specialized concave structure (pollen basket) that carries pollen (Michener 1999), whereas a scopa is a setal structure that carries pollen and may be located on the abdomen or tibia. Kleptoparasitic bees do not have pollen collection structures. |
| Flight phenology | 1: Early  2: Middle  3: Late | Early phenology was defined as peak abundance in May and June. Mid-season phenology was defined as peak abundance in late June through July. Late-season phenology was defined as peak abundance in August. |
| Sociality | 1: Solitary  2: Social  3: Multiple  4: Kleptoparasite | In solitary bees, females do everything required to raise offspring including excavating/renting nests, laying eggs and provisioning larvae (Linsely 1958, Danforth et al. 2019). Social bees divide labor of among queens, drones, and workers. Social bees typically have a higher foraging and reproductive capacity and have a faster response to resource provision than solitary bees (Michener 2007). ‘Multiple’ refers to bee species which exhibit both solitary and primitive communal behaviors, and kleptoparasites are typically solitary bees that rob nests of other bees. |
| Body size (mm) | Intertegular distance (ITD) | ITD is the distance between the inner nodes of the wing base. ITD is a proxy of dry weight and foraging distance (Greenleaf and Kremen 2006) and tongue length (Cariveau et al. 2016). |
