# Supplementary material for: Livestock grazing is associated with seasonal reduction in pollinator biodiversity and functional dispersion but cheatgrass invasion is not: Variation in bee assemblages in a multi-use shortgrass prairie
Source: PLoS One. 2020 Dec 17;15(12):e0237484. doi: 10.1371/journal.pone.0237484 (PMC7746148; doi:10.1371/journal.pone.0237484)
Supplement: S2 Table — For continuous variables (ITD), values are the mean from n = 10 specimens. (DOCX) [file pone.0237484.s002.docx]

| **Family** | **Genus** | **species** | **ITD** | **Nest**  **location** | **Nest**  **construction** | **Sociality** | **Lecty** | **Pollen**  **collection**  **structure** | **Tibial hair**  **density** | **Flight**  **phenology** |
| --- | --- | --- | --- | --- | --- | --- | --- | --- | --- | --- |
| Apidae | *Anthophora* | *affabilis* | 4.421 | below | excavate | solitary | poly | scopa | high | middle |
|  |  | *bomboides* | 3.694 | below | excavate | solitary | poly | scopa | high | early |
|  |  | *montana* | 3.694 | below | excavate | solitary | poly | scopa | high | middle |
|  |  | *occidentalis* | 4.205 | below | excavate | solitary | poly | scopa | high | middle |
|  | *Apis* | *mellifera* | 5.012 | above | excavate | social | poly | corbicula | low | middle |
|  | *Bombus* | *appositus* | 7.474 | below | rent | social | poly | corbicula | low | middle |
|  |  | *bifarius* | 5.186 | below | rent | social | poly | corbicula | low | late |
|  |  | *californicus* | 5.964 | below | rent | social | poly | corbicula | low | middle |
|  |  | *centralis* | 3.379 | below | rent | social | poly | corbicula | low | middle |
|  |  | *fervidus* | 7.824 | below | rent | social | poly | corbicula | low | middle |
|  |  | *griseocollis* | 7.780 | below | rent | social | poly | corbicula | low | late |
|  |  | *huntii* | 7.140 | below | rent | social | poly | corbicula | low | late |
|  |  | *insularis* | 5.453 | klepto | klepto | klepto | klepto | klepto | low | early |
|  |  | *nevadensis* | 4.940 | below | rent | social | poly | corbicula | low | early |
|  |  | *pensylvanicus* | 7.426 | below | rent | social | poly | corbicula | low | late |
|  |  | *rufocinctus* | 4.87 | below | rent | social | poly | corbicula | low | middle |
|  |  | *sylvicola* | 5.55 | below | rent | social | poly | corbicula | low | late |
|  | *Diadasia* | *enavata* | 4.38 | below | excavate | solitary | oligo | scopa | high | middle |
|  | *Eucera* | *hamata* | 2.65 | below | excavate | solitary | poly | scopa | high | early |
|  |  | *lepida* | 3.86 | below | excavate | solitary | poly | scopa | high | early |
|  | *Melecta* | *pacifica* | 3.42 | klepto | klepto | klepto | klepto | klepto | low | early |
|  | *Melissodes* | *agilis* | 3.81 | below | excavate | solitary | oligo | scopa | high | late |
|  |  | *communis* | 3.98 | below | excavate | solitary | oligo | scopa | high | middle |
|  |  | *coreopsis* | 3.44 | below | excavate | solitary | oligo | scopa | high | middle |
|  |  | *sp 1* | 3.56 | below | excavate | solitary | oligo | scopa | high | middle |
|  |  | *tristis* | 3.19 | below | excavate | solitary | oligo | scopa | high | late |
|  | *Svastra* | *obliqua* | 3.88 | below | excavate | solitary | oligo | scopa | high | late |
|  |  | *petulca* | 3.60 | below | excavate | solitary | oligo | scopa | high | middle |
|  | *Xeromelecta* | *interrupta* | 2.98 | klepto | klepto | klepto | klepto | klepto | low | middle |
| Colletidae | *Colletes* | *sp 1* | 3.04 | below | excavate | solitary | poly | scopa | medium | middle |
| Halictidae | *Agapostemon* | *angelicus* | 2.56 | below | excavate | multiple | poly | scopa | medium | late |
|  |  | *coloradinus* | 2.56 | below | excavate | multiple | poly | scopa | medium | middle |
|  |  | *texanus* | 2.91 | below | excavate | multiple | poly | scopa | medium | late |
|  |  | *virescens* | 3.48 | below | excavate | multiple | poly | scopa | medium | middle |
|  | *Augochlorella* | *aurata* | 2.03 | below | excavate | social | poly | scopa | medium | late |
|  | *Lasioglossum* | *dialictus* | 1.13 | below | excavate | multiple | poly | scopa | medium | middle |
|  |  | *sp 1* | 1.84 | below | excavate | multiple | poly | scopa | medium | early |
|  | *Halictus* | *sp 1* | 1.42 | below | excavate | multiple | poly | scopa | medium | middle |
|  |  | *ligatus* | 1.75 | below | excavate | multiple | poly | scopa | medium | middle |
|  |  | *tripartitus* | 1.10 | below | excavate | multiple | poly | scopa | medium | middle |
| Megachilidae | *Anthidium* | *sp 1* | 3.26 | klepto | klepto | solitary | klepto | klepto | low | middle |
|  | *Lithurgopsis* | *apicalis* | 3.64 | above | rent | multiple | oligo | abdomen | low | late |
|  | *Megachile* | *dentitarsus* | 4.24 | above | rent | solitary | poly | abdomen | low | middle |
|  |  | *sp1* | 2.77 | above | rent | solitary | poly | abdomen | low | middle |
|  |  | *sp2* | 3.75 | above | rent | solitary | poly | abdomen | low | middle |
|  |  | *sp 3* | 4.28 | above | rent | solitary | poly | abdomen | low | middle |
|  | *Osmia* | *sp1* | 2.73 | above | rent | solitary | poly | scopa | medium | early |
|  |  | *sp2* | 2.10 | above | rent | solitary | poly | scopa | medium | early |
|  |  | *sp 3* | 2.40 | above | rent | solitary | poly | scopa | medium | late |
