# Supplementary material for: Livestock grazing is associated with seasonal reduction in pollinator biodiversity and functional dispersion but cheatgrass invasion is not: Variation in bee assemblages in a multi-use shortgrass prairie
Source: PLoS One. 2020 Dec 17;15(12):e0237484. doi: 10.1371/journal.pone.0237484 (PMC7746148; doi:10.1371/journal.pone.0237484)
Supplement: S3 Table — Months in which abundances were maximized were used to assign estimates of bee phenology for functional trait analysis. ‘Early-season’ = May or June; ‘Mid-season’ = July, and ‘Late-season’ = August. Cells are highlighted in gray to denote peak month of capture for each species. (DOCX) [file pone.0237484.s003.docx]

| **Family** | **Genus** | **species** | **Month** | | | |
| --- | --- | --- | --- | --- | --- | --- |
|  |  |  | May | June | July | August |
| Apidae | *Anthophora* | *affabilis* | 43 | 54 | 10 | 1 |
|  |  | *bomboides* | 2 | 3 | 2 | 0 |
|  |  | *montana* | 14 | 49 | 17 | 42 |
|  |  | *occidentalis* | 0 | 13 | 88 | 7 |
|  | *Apis* | *mellifera* | 6 | 54 | 19 | 12 |
|  | *Bombus* | *appositus* | 6 | 40 | 75 | 34 |
|  |  | *bifarius* | 0 | 0 | 1 | 1 |
|  |  | *californicus* | 0 | 2 | 0 | 0 |
|  |  | *centralis* | 0 | 2 | 2 | 0 |
|  |  | *fervidus* | 3 | 87 | 112 | 51 |
|  |  | *griseocollis* | 2 | 6 | 20 | 18 |
|  |  | *huntii* | 11 | 3 | 15 | 22 |
|  |  | *insularis* | 1 | 0 | 0 | 0 |
|  |  | *nevadensis* | 71 | 177 | 38 | 2 |
|  |  | *pensylvanicus* | 10 | 68 | 191 | 212 |
|  |  | *rufocinctus* | 1 | 7 | 2 | 3 |
|  |  | *sylvicola* | 0 | 2 | 5 | 4 |
|  | *Diadasia* | *enavata* | 0 | 26 | 2 | 1 |
|  | *Eucera* | *hamata* | 82 | 22 | 0 | 0 |
|  |  | *lepida* | 4 | 0 | 0 | 0 |
|  | *Melecta* | *pacifica* | 27 | 1 | 0 | 0 |
|  | *Melissodes* | *agilis* | 0 | 1 | 64 | 194 |
|  |  | *communis* | 7 | 170 | 153 | 66 |
|  |  | *coreopsis* | 0 | 19 | 38 | 24 |
|  |  | sp. 1 | 0 | 8 | 4 | 3 |
|  |  | *tristis* | 1 | 15 | 94 | 162 |
|  | *Svastra* | *obliqua* | 0 | 4 | 146 | 93 |
|  |  | *petulca* | 0 | 0 | 11 | 7 |
|  | *Xeromelecta* | *interrupta* | 1 | 22 | 2 | 1 |
| Colletidae | *Colletes* | sp. 1 | 0 | 1 | 1 | 0 |
| Halictidae | *Agapostemon* | *angelicus* | 5 | 11 | 23 | 14 |
|  |  | *coloradinus* | 0 | 6 | 3 | 2 |
|  |  | *texanus* | 2 | 2 | 41 | 23 |
|  |  | *virescens* | 15 | 48 | 10 | 5 |
|  | *Augochlorella* | *aurata* | 3 | 4 | 4 | 14 |
|  | *Halictus* | sp. 1 | 17 | 36 | 21 | 3 |
|  |  | *ligatus* | 2 | 2 | 14 | 2 |
|  |  | *tripartitus* | 8 | 105 | 203 | 33 |
|  | *Lasioglossum* | *dialictus* | 37 | 108 | 48 | 39 |
|  | *Lasioglossum* | sp. 1 | 11 | 15 | 5 | 2 |
| Megachilidae | *Anthidium* | sp. 1 | 0 | 3 | 7 | 3 |
|  | *Lithurgopsis* | *apicalis* | 0 | 6 | 16 | 4 |
|  | *Megachile* | *dentitarsus* | 0 | 1 | 2 | 1 |
|  |  | sp. 1 | 3 | 7 | 3 | 6 |
|  |  | sp. 2 | 6 | 32 | 28 | 20 |
|  |  | sp. 3 | 0 | 2 | 1 | 1 |
|  | *Osmia* | sp. 1 | 23 | 2 | 1 | 0 |
|  |  | sp. 2 | 14 | 4 | 2 | 0 |
|  |  | sp. 3 | 1 | 0 | 1 | 2 |
