# Supplementary material for: Livestock grazing is associated with seasonal reduction in pollinator biodiversity and functional dispersion but cheatgrass invasion is not: Variation in bee assemblages in a multi-use shortgrass prairie
Source: PLoS One. 2020 Dec 17;15(12):e0237484. doi: 10.1371/journal.pone.0237484 (PMC7746148; doi:10.1371/journal.pone.0237484)
Supplement: S4 Table — Significant and marginally significant parameters are highlighted in bold text. (DOCX) [file pone.0237484.s004.docx]

| **Response** | **Parameter** | **β** | **SE** | ***t-*score** | ***P*** |
| --- | --- | --- | --- | --- | --- |
| Bee abundance | (Intercept) | -0.007 | 0.166 | -0.040 | 0.968 |
|  | **floral cover** | **0.549** | **0.243** | **2.255** | **0.037** |
|  | floral richness | -0.513 | 0.322 | -1.596 | 0.128 |
|  | bareground cover | 0.221 | 0.251 | 0.881 | 0.390 |
|  | litter/wood cover | -0.219 | 0.304 | -0.719 | 0.481 |
|  | cheatgrass cover | -0.487 | 0.520 | -0.935 | 0.362 |
|  | grass cover | -0.811 | 0.491 | -1.650 | 0.116 |
|  | rock cover | -0.096 | 0.186 | -0.518 | 0.611 |
|  |  |  |  |  |  |
| Bee richness | (Intercept) | 0.011 | 0.175 | 0.062 | 0.951 |
|  | floral cover | 0.298 | 0.257 | 1.162 | 0.261 |
|  | floral richness | -0.209 | 0.340 | -0.617 | 0.545 |
|  | bareground cover | 0.232 | 0.265 | 0.874 | 0.394 |
|  | litter/wood cover | 0.056 | 0.321 | 0.175 | 0.863 |
|  | cheatgrass cover | -0.066 | 0.549 | -0.119 | 0.906 |
|  | grass cover | -0.712 | 0.518 | -1.373 | 0.187 |
|  | rock cover | -0.292 | 0.196 | -1.488 | 0.154 |
|  |  |  |  |  |  |
| α-diversity | (Intercept) | 0.002 | 0.185 | 0.013 | 0.990 |
|  | floral cover | 0.123 | 0.271 | 0.454 | 0.655 |
|  | floral richness | 0.002 | 0.358 | 0.005 | 0.996 |
|  | bareground cover | 0.150 | 0.280 | 0.536 | 0.598 |
|  | litter/wood cover | 0.299 | 0.339 | 0.883 | 0.389 |
|  | cheatgrass cover | 0.221 | 0.580 | 0.381 | 0.708 |
|  | grass cover | -0.428 | 0.547 | -0.782 | 0.444 |
|  | rock cover | -0.240 | 0.207 | -1.162 | 0.260 |
|  |  |  |  |  |  |
| Functional | (Intercept) | 0.030 | 0.146 | 0.206 | 0.839 |
| dispersion | floral cover | -0.293 | 0.215 | -1.367 | 0.188 |
|  | floral richness | -0.170 | 0.284 | -0.599 | 0.556 |
|  | **bareground cover** | **-0.673** | **0.221** | **-3.041** | **0.007** |
|  | litter/wood cover | 0.020 | 0.268 | 0.076 | 0.940 |
|  | cheatgrass cover | -0.704 | 0.459 | -1.535 | 0.142 |
|  | **grass cover** | **-0.848** | **0.433** | **-1.958** | **0.066** |
|  | rock cover | 0.166 | 0.164 | 1.016 | 0.323 |
