# Supplementary figures and images for: Livestock grazing is associated with seasonal reduction in pollinator biodiversity and functional dispersion but cheatgrass invasion is not: Variation in bee assemblages in a multi-use shortgrass prairie
Source: PLoS One. 2020 Dec 17;15(12):e0237484. doi: 10.1371/journal.pone.0237484 (PMC7746148; doi:10.1371/journal.pone.0237484)

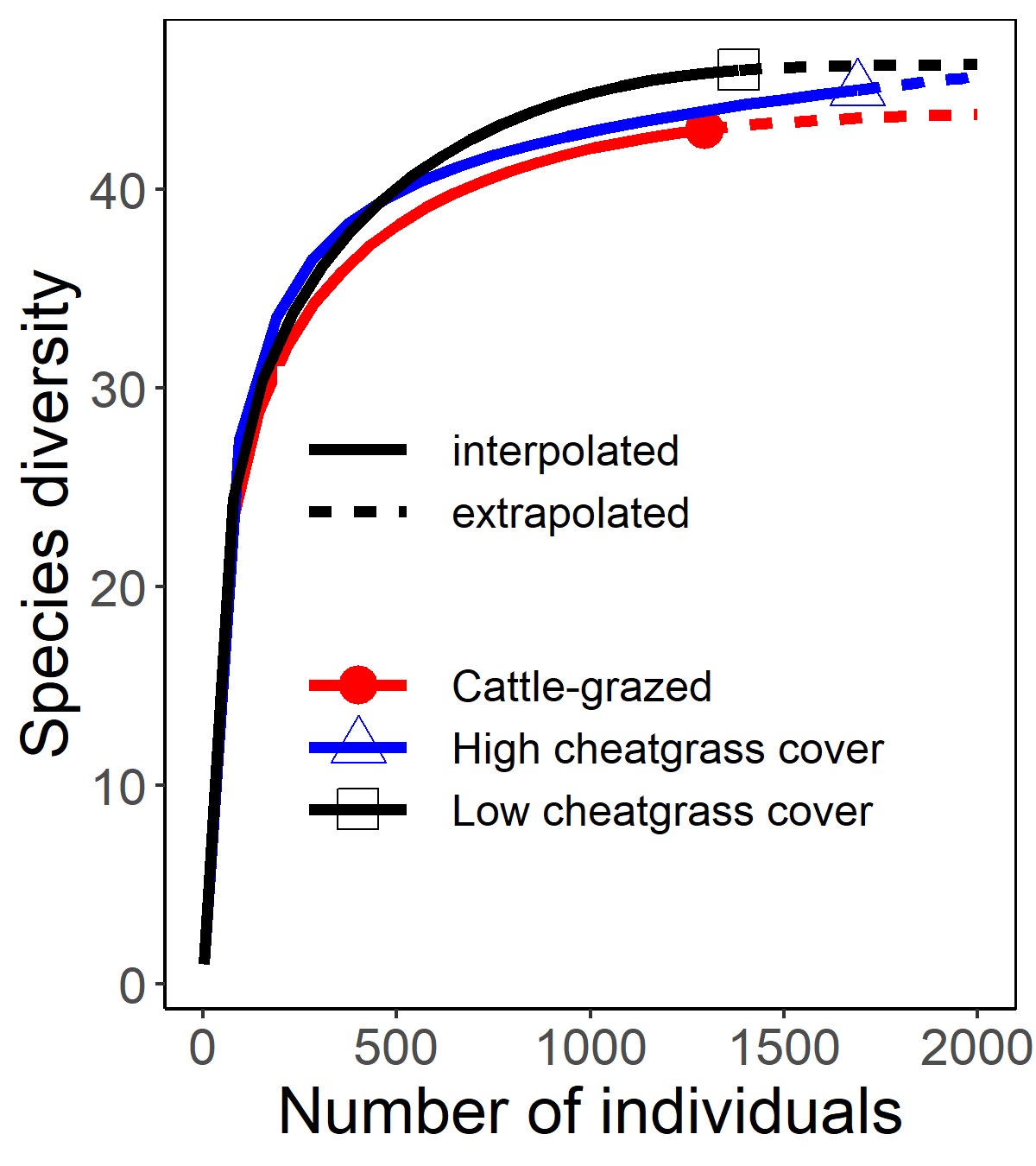


**S1 Fig. Species detection curves for wild bees in shortgrass prairie in three habitat types.**

Supplement: S1 Fig — (DOCX) [file pone.0237484.s005.docx]
